# Metabolomics Characterization of Two Apocynaceae Plants, *Catharanthus roseus* and *Vinca minor*, Using GC-MS and LC-MS Methods in Combination

**DOI:** 10.3390/molecules22060997

**Published:** 2017-06-17

**Authors:** Qi Chen, Xueyan Lu, Xiaorui Guo, Qingxi Guo, Dewen Li

**Affiliations:** 1 Center for Ecological Research, Northeast Forestry University, Harbin 150040, China; 18304626637@163.com (Q.C.); nefu20064764@126.com (X.L.); 2 Key Laboratory of Plant Ecology, Northeast Forestry University, Harbin 150040, China; xruiguo@nefu.edu.cn

**Keywords:** *Catharanthus roseus*, *Vinca minor*, GC-MS, LC-MS, metabolomics, TIAs

## Abstract

*Catharanthus roseus* (*C. roseus*) and *Vinca minor* (*V. minor*) are two common important medical plants belonging to the family Apocynaceae. In this study, we used non-targeted GC-MS and targeted LC-MS metabolomics to dissect the metabolic profile of two plants with comparable phenotypic and metabolic differences. A total of 58 significantly different metabolites were present in different quantities according to PCA and PLS-DA score plots of the GC-MS analysis. The 58 identified compounds comprised 16 sugars, eight amino acids, nine alcohols and 18 organic acids. We subjected these metabolites into KEGG pathway enrichment analysis and highlighted 27 metabolic pathways, concentrated on the TCA cycle, glycometabolism, oligosaccharides, and polyol and lipid transporter (RFOS). Among the primary metabolites, trehalose, raffinose, digalacturonic acid and gallic acid were revealed to be the most significant marker compounds between the two plants, presumably contributing to species-specific phenotypic and metabolic discrepancy. The profiling of nine typical alkaloids in both plants using LC-MS method highlighted higher levels of crucial terpenoid indole alkaloid (TIA) intermediates of loganin, serpentine, and tabersonine in *V. minor* than in *C. roseus*. The possible underlying process of the metabolic flux from primary metabolism pathways to TIA synthesis was discussed and proposed. Generally speaking, this work provides a full-scale comparison of primary and secondary metabolites between two medical plants and a metabolic explanation of their TIA accumulation and phenotype differences.

## 1. Introduction

*Catharanthus roseus* and *Vinca minor* are evergreen perennial plants belonging to the family Apocynaceae, well known as medicinal and ornamental plants around the world [1,2]. Their main metabolites, terpenoid indole alkaloids (TIAs), are widely used to treat human diseases [3]. More than 50 TIAs have been isolated from *V. minor* plants [4]. The most widely used TIA is vincamine, which has modulatory effects on brain circulation and neuronal homeostasis, as well as antihypoxic and neuroprotective potencies. It has been used for the prevention and treatment of cerebrovascular insufficiencies and a variety of cerebral disorders; it is also as an active ingredient in dietary supplements used as smart drugs, cognitive enhancers or nootropics [3,5,6]. *C. roseus*, originally a species endemic to Madagascar, has become an important model plant system for medical, biotechnology and secondary metabolism studies [7]. *C. roseus* is a remarkable source of secondary metabolites, with more than 130 TIAs having already been described [8]. Many of the TIAs are anticancer agents, which includes ajmalicine, catharanthine, serpentine, vindoline, vinblastine, and vincristine [9]. Vinblastine is an efficient inhibitor of microtubule polymerization and is used to treat several forms of cancer, (e.g., Hodgkin’s disease and, malignant lymphoma), and a wide variety of other human neoplasms [9,10]. *V. minor* and *C. roseus* plants have been reported to contain a substantial variety of common TIA intermediates, especially from upstream pathways. However, the similarity between the *V. minor* and *C. roseus* is currently unclear.

Leaves are the main sites for TIA accumulation, and have significant differences in morphology between the two plant species: *V. minor* ones are leathery, oblong to ovate, while *C. roseus* leaves are membranous, and obovate-oblong. This phenotypic discrepancy provides a hint about the metabolic and adaptive differences existing between them [11,12]. The leaf development and phenotype are generally under genetic and environmental controls, reflecting metabolite status such as the ratio of carbohydrate to acids levels [12]. The Sugar (Carbohydrate) ratio could affect the growth of plants, regulate environmental responses and play a role in signal transduction [12,13]. There is a close and complex link between sugar levels and growth processes of plant development [14]. The organ formation and leaf morphology in plants could also be regulated by carbohydrate signaling [15]. It has been reported that leaves of *Arabidopsis* couldn’t extend under high levels of sugar [16]. Gene overexpression studies revealed that excessive accumulation of trehalose in *A. thaliana* could lead to stunted growth, early flowering and increased shoot formation [17,18]. It also has been noted that embryo rescue by dexamethasone-inducible or embryo-specific expression of AtTPS1, null tps1 mutants grow very slowly and are almost infertile [13,19]. Increased Tre6P levels lead to lower tuber size and yield, in growing potato tubers [4]. Furthermore, among the numerous saccharide compounds, raffinose is significantly related to plant stress hardiness [20]. Raffinose is found to be able to increase cold tolerance during the process of cold acclimation [21]. Raffinose content was reported to be slightly increased by chilling treatment at 13/10 °C for 4 days in cold-tolerant rice [22].

Acids are another large class of metabolites in plants and considered as a very important barrier layer of leaves as a defense against biotic and abiotic damages. Among them, phenolic the chlorogenic acids are widely distributed in plant kingdom, displaying notable antibacterial properties [23,24]. This antibacterial activity was previously revealed by disc diffusion tests in *Centella asiatica*, artichoke and burdock [25,26,27]. In addition, studies have indicated that chlorogenic acid effectively inhibited the growth of all tested bacterial pathogens (*Shigella dysenteria* and *S. pneumoniae*), and it was effective against both Gram-negative and Gram-positive bacteria as described by Lou [23].

To reveal the link between metabolic profiling and its silent phenotypic characterization, we used metabolomic strategies to dissect their independent systems biology traits [28]. GC-MS and LC-MS have been proved to be powerful tools for plant metabolomics research, especially facilitating the identification and quantification of those metabolites involved in the central pathways of metabolism [28,29]. In this study, we applied them in combination to profiling comparable metabolic profiles in leaves of *C. roseus* and *V. minor*. The investigated targets include basic morphological and physiological parameters, non-targeted primary metabolites and targeted secondary metabolites. We attempted to not only provide an overview of metabolic network differences, but also search for crucial components contributing to their phenotypic and physiological performance.

## 2. Results

### 2.1. Leaf Morphology Characteristics

**Figure 1:**
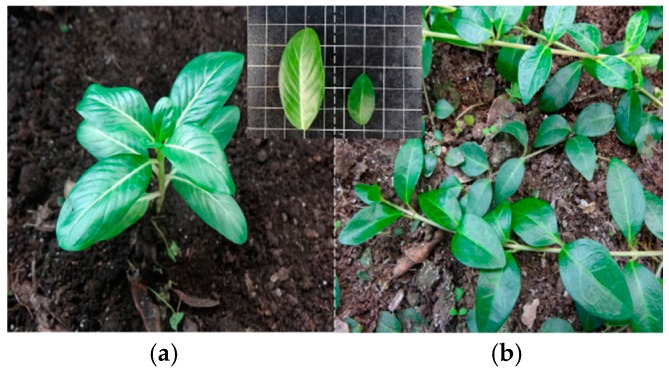
Leaf phenotypes of *Catharanthus roseus* and *Vinca minor*. (**a**) *C. roseus*; (**b**) *V. minor*.

As shown in Figure 1, *C. roseus* is an upright shrub with obovate-oblong leaves, while *V. minor* is herbaceous chamaephyta scandentia with obround to oval leaves. The leaves of *V. minor* are smaller in size, deeper in color and much more leathery compared to those of *C. roseus*. The leaf length (LL), leaf width (LW), leaf area (LA) and fresh weight (FW) values in *V. minor* were only close to 1/2–1/3 those of *C. roseus* (Table 1). However, the leaf mass per unit area (LMA) and leaf thickness (LT) values of *V. minor* were significantly (1.5-fold and 1.7-fold) bigger than in *C. roseus*, respectively (Table 1). In addition, *V. minor* plants normally grow in temperate zones and their preferred temperature ranges from 15 °C to 25 °C, while *C. roseus* was originally distributed in the torrid-zone with a preferred temperature from 20 °C to 33 °C. *C. roseus* plants have a higher response to photosynthesis (Table 1). Comparing leaf photosynthesis-related parameters, we found that *C. roseus* has higher levels of Y(II), net photosynthesis rate (Pn) and Fv/Fm, and lower levels of chlorophyll (Chl), chlorophyll a/chlorophyll b (Chla/Chlb), Transpiration rate(Tr), relative water content (RWC) and stomatal conductance (Gs) than *V. minor* (Table 1).

### 2.2. Primary Metabolic Profiling

**Figure 2:**
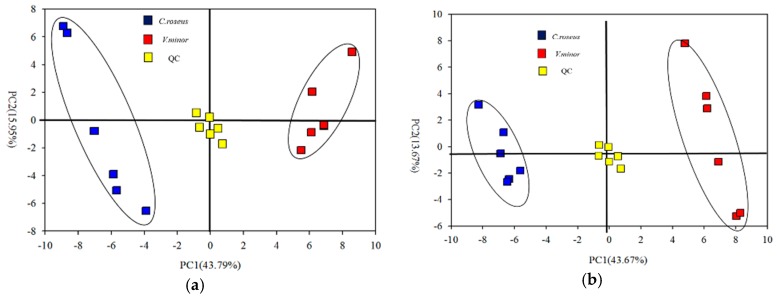
The multivariate analysis of primary metabolites in *Catharanthus roseus* and *Vinca minor*. (**a**) PCA score plot; (**b**) PLS-DA score plot. Blue indicates *C. roseus*, red indicates *V. minor*, and yellow indicates Quality Control.

**Figure 3:**
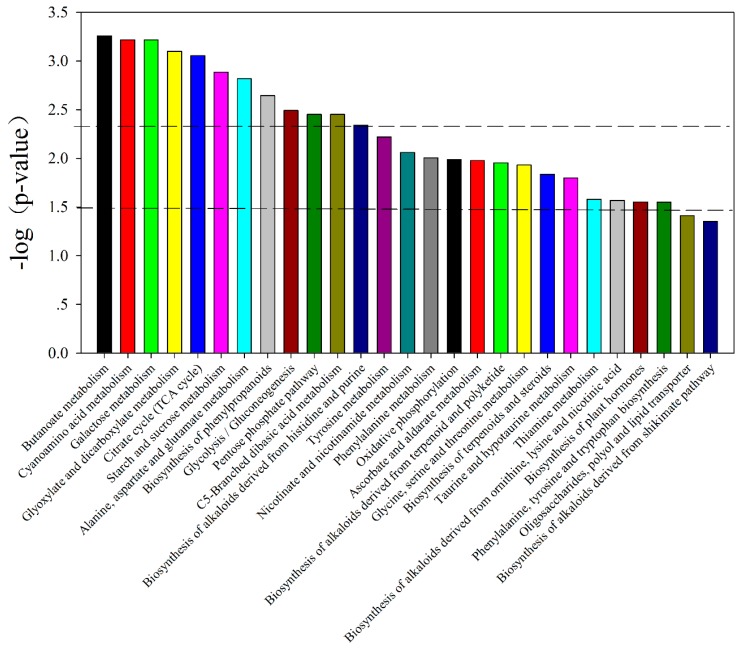
The histogram of different pathway −log (*p*-value, 10). The horizontal line of 1.3 indicates *p* < 0.05, the horizontal line of 1.5 indicates *p* < 0.01.

For displaying data structure and species specificity between *C. roseus* and *V. minor* leaves, we reduced the dimensionality of the data and visualized samples groupings. An unsupervised multivariate data analysis method (PCA) was applied on the GC-MS data generated from the *C. roseus* and *V. minor* groups (Figure 2a). Regarding the PCA score plot, the two species showed significant separations, with values of 43.79% and 15.95%, respectively (Figure 2a). PLS-DA is a supervised method, which could classify the observations in groups giving the largest predicted indicator variable, and the predictability values are 43.67% and 13.67% (Figure 2b) (model validation is shown in Figure S1). A total of 58 significant metabolites were obtained according to their VIP values (VIP > 1) and *p*-values (*p* < 0.05), false discovery rate (FDR, (FDR < 0.05)) from 116 compounds in the GC-MS data (Table 2, Table S8). These metabolites displayed substantial differences in abundance in the leaves of *C. roseus* and *V. minor* (Table 2). All the different metabolites were annotated to the biological pathways listed in the KEGG database, and assigned to 27 pathways selected by *p* < 0.05 (Figure 3). There were nine kinds of sugars (from a total of 16), five kinds of amino acids (eight in total), six kinds of alcohol (total was nine), and six kinds of organic acids (from a total of 18) that were found to be significantly less accumulated in *V. minor* compared to *C. roseus* (Table 2).

**Figure 4:**
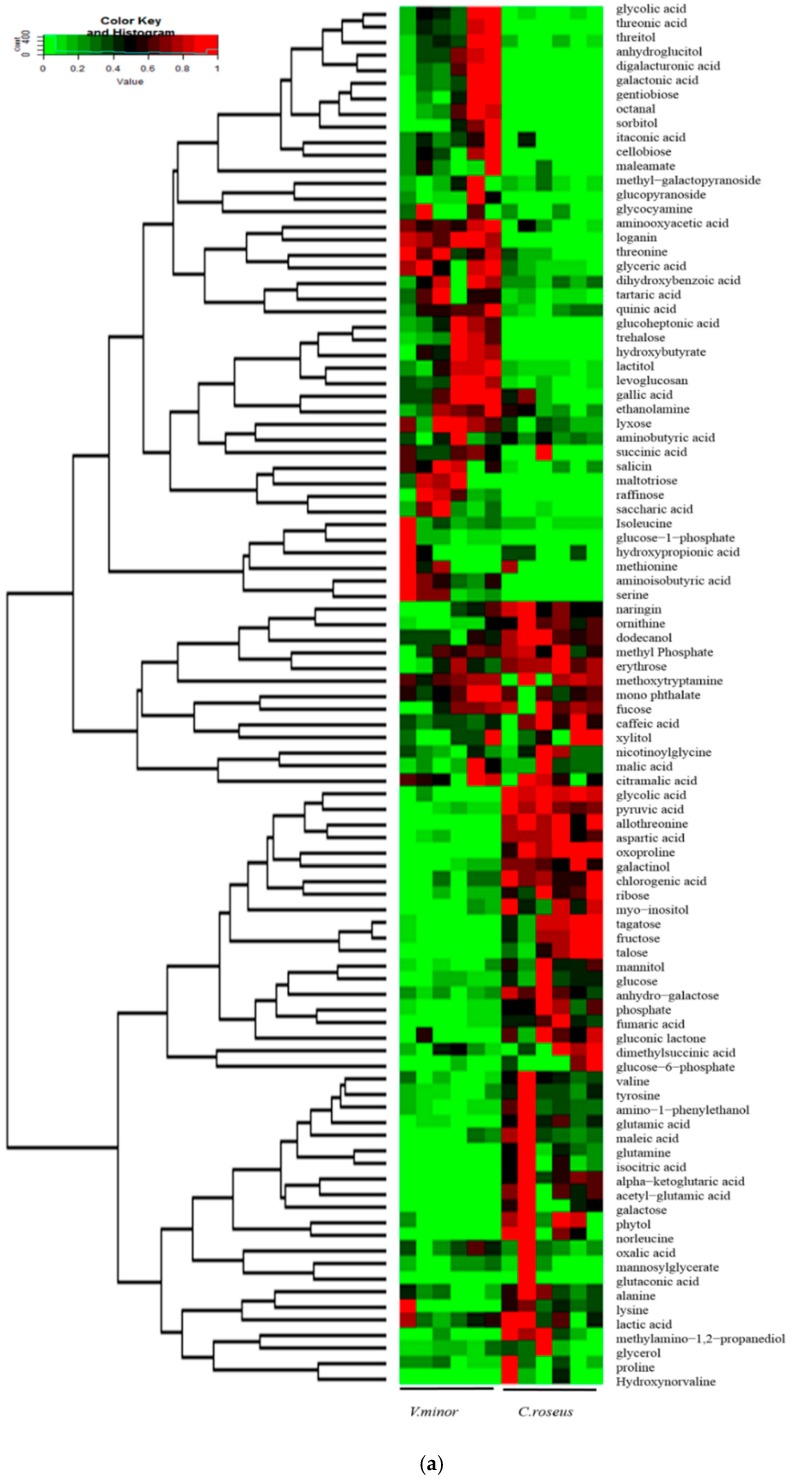

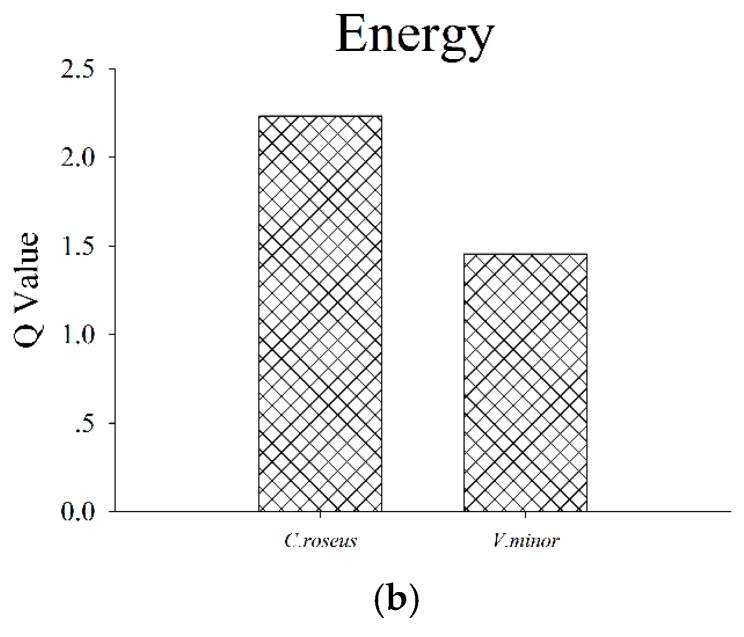
The cluster of metabolites and energy *Q* values between *Catharanthus roseus* and *Vinca minor*. (**a**) Heat map visualization of relative differences of metabolites in *C. roseus* and *V. minor*. The content value of each metabolite was normalized to complete linkage hierarchical clustering. The metabolites were glycolic acid, threonic acid, threitol, anhydroglucitol, digalacturonic acid, galactonic acid, gentiobiose, octanal, sorbitol, itaconic acid, cellobiose, maleamate, methyl- galactopyranoside, glucopyranoside, glycocyamine, aminooxyacetic acid, loganin, threonine, threonine, glyceric acid, dihydroxybenzoic acid, tartaric acid, quinic acid, glucoheptonic acid, trehalose, hydroxybutyrate, lactitol, levoglucosan, gallic acid, ethanolamine, lyxose, aminobutyric acid, succinic acid, salicin, maltotriose, raffinose, saccharic acid, isoleucine, lucose-1-phosphate, hydroxypropionic acid, methionine, aminoisobutyric acid, serine, ornithine, dodecanol, methyl phosphate, erythrose, methoxytryptamine, mono phthalate, fucose, caffeic acid, xylitol, nicotinoylglycine, malic acid, citramalic acid, glycolic acid, pyruvic acid, allothreonine, aspartic acid, oxoproline, galactinol, chlorogenic acid, ribose, myo-inositol, tagatose, fructose, talose, mannitol, glucose, anhydrogalactose, phosphate, fumaric acid, gluconic lactone, dimethylsuccinic acid, glucose-6-phosphate, valine, tyrosine, amino-1-phenylethanol, glutamic acid, maleic acid, glutamine, isocitric acid, alpha-ketoglutaric acid, acetyl-glutamic acid, galactose, phytol, norleucine, oxalic acid, mannosylglycerate, glutaconic acid, alanine, lysine, lactic acid, methyl-amino-1,2-propanediol, glycerol, proline, hydroxynorvaline, from top to bottom, in turn. Red indicates high abundance, whereas low relative metabolites are green. V1, V2, V3, V4, V5, V6 indicate *V. minor*, C1, C2, C3, C4, C5, C6 indicate *C. roseus*; (**b**) The Q value of energy between *Catharanthus roseus* and *Vinca minor*.

**Figure 5:**
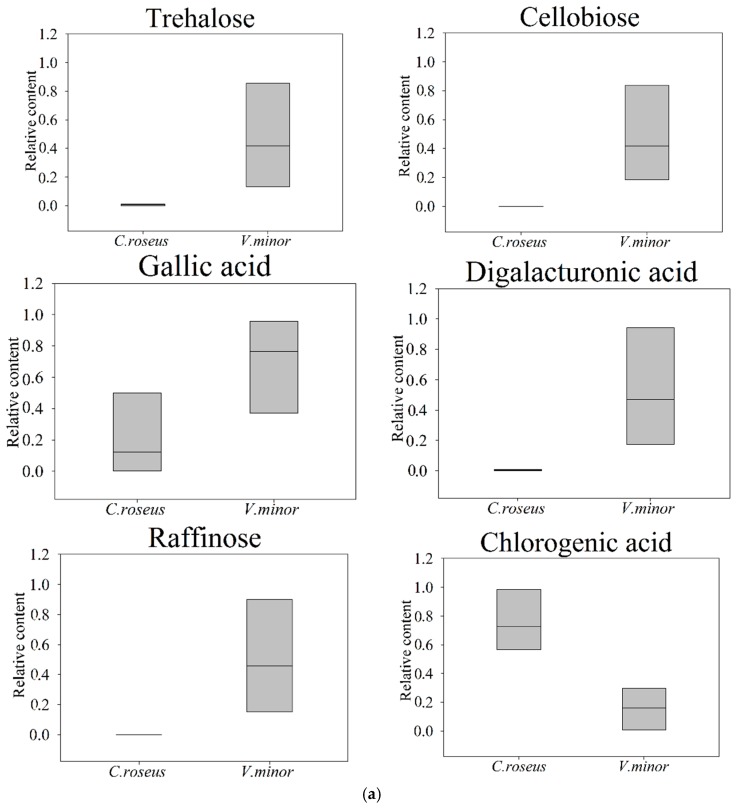

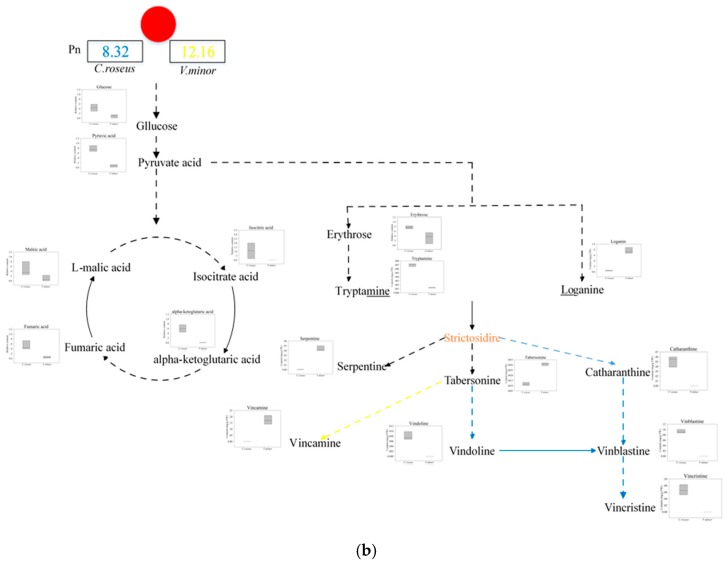
The relative content of major different metabolites and biochemical pathway map. (**a**) Relative content of trehalose, cellobise, gallic acid, digalacturonic acid, raffinose and chlorogenic acid. (**b**) Visualization of secondary metabolites, photosynthesis and TCA cycle in a biochemical pathway map. Common metabolites are written black, *V. minor* ones are written in yellow, and red metabolites represent no content to display, *C. roseus* are written in blue, full lines represent one step reactions, and dashed lines represent multi-step reactions.

The results of hierarchical cluster analysis and heat map were illustrated in Figure 4a and Table 2, respectively. There are more energy sources in *C. roseus* than *V. minor* based on energy the *Q* value (Figure 4). Among them, we found that cellobiose, raffinose and octanal were only present in *V. minor* leaves. Amino acids and sugars were mainly accumulated in *C. roseus*, while organic acids were largely accumulated in *V. minor* (Table 2). The contents of TCA cycle intermediates in *V. minor* were significantly low: fumaric acid accounted for only 8.69%, α-ketoglutaric acid accounted for 15.8% and pyruvic acid counted for 9.48%, compared with *C. roseus* (Table 2). The compound raffinose appeared only in *V. minor* (Figure 5a).

The other marker metabolites, such as myo-inositol, galactinol, fructose and glucose, were significantly less accumulated in *V. minor* (Table 2). Trehalose (78-fold) and cellobiose (only in *V. minor*) belonging to the starch and sucrose metabolism were markedly accumulated in *V. minor* (Figure 5a and Table 2). The other metabolites related to leaf development have a high content in *V. minor*, for example gallic acid (3-fold) and galactonic acid (19-fold) compared with *C. roseus* (Figure 5a, Table 2). The results showed that the TCA cycle, including galactose metabolism, starch and sucrose metabolism and oligosaccharides, polyol and lipid transporter pathways were closely associated with energy, leaf development and morphology (Figure 5a, Table 2). Except for sorbitol/mannitol, the high accumulations of oligosaccharides, polyol and lipid transporter metabolites suggested that a frequent transportation of them existed in *V. minor* (Table 2).

### 2.3. The Content of TIAs

The leaves in plants are known to play a critical role in the TIAs synthesis pathway. Loganin, tryptamine, tabersonine, serpentine, vindoline, catharanthine, vinblastine, vincristine and vincamine were the major and representative TIAs in *C. roseus* and *V. minor*, respectively. This enabled us to conduct a comparison between plant relatedness and the number of shared metabolites. The *V. minor* leaves were found to have higher Pn rate and highly accumulated metabolites belonging to the direct glycogen and TCA cycle, compared with *C. roseus* (Figure 5b).

Regarding the contents of TIAs detected in the two species, we found that the V. minor leaves accumulated higher levels of loganin (17.6-fold), tabersonine (2.7-fold) and serpentine (117.9-fold), and a lower level of tryptamine than did *C. roseus* (Figure 5, Table S7). The levels of species-specific compounds, including vincamine in *V. minor*, and vindoline, catharanthine, vinblastine and vincristine in *C. roseus*, are also shown in Figure 5b.

## 3. Discussion

There are enormous differences in leaf morphology between *V. minor* and *C. roseus*. In this study, we utilized the widely non-targeted GC-MS method for the direct chemical screening of the primary metabolites related to morphology and development. Then, we used the targeted LC-MS method to compare the levels of the species-specific TIAs in the two species.

The evolutional phenotypic plasticity of the leaf accelerates plant adaptation to the environment. There is a tight correlation between plant metabolism and morphology and it was reported that the leaf morphology could be affected by specific metabolites [30,31]. The main primary metabolite sugars, such as trehalose, sucrose and fructose, are considered important signaling molecules, regulating the carbon status and starch biosynthesis in plants [30]. In our study, some characteristic sugars that could affect the morphology of leaf were detected, displaying significant species-specific accumulation pattern (Table 2). Trehalose is actively associated with the physiological parameters of plant leaves and limits the growth and development of leaves [32]. Trehalose has been suggested as a regulatory component in the control of glycolytic flux and in a variety of stress survival strategies [30]. Plants overaccumulating trehalose have significantly retarded growth. Studies with *A. thaliana 35S::otsA* lines generating higher Tre6P than wild-type plants displayed a lower specific leaf area and dry weight content per unit fresh weight [30,31]. Tobacco plants over-expressing trehalose phosphate synthase (TPS) exhibited stunted growth, developing small, dark, lancet-shaped leaves with increased photosynthetic capacity and delayed senescence [33,34,35]. There is also direct experimental evidence that overexpression of trehalose-6-phosphate phosphatase (Tpp) could limit the growth of *Arabidopsis* leaves [30,35]. These observations were confirmed in our experimental results, showing that *V. minor* simultaneously has a higher relative content of trehalose and a smaller leaf area.

In addition, trehalose is proved to play a role in regulating the rate of starch degradation according to the demand for sucrose [36]. It was shown to inhibit the SNF1-related protein kinase, thereby affecting the consumption of sucrose by restricting growth [14,30,37]. In addition, the carbon (the skeleton of sugars) use of plant was also dependent on signal sugar status, and carbohydrate metabolites was closely related to the morphological differences in leaves [30,31]. Therefore, based on our experimental results, we speculate that trehalose might be the major contributor to the observed discrepancies in leaf morphology and composition.

In our observation, the leaves of *V. minor* were thicker, harder and leathery. Combined with the biological characteristics of metabolites, we hypothesized that some metabolic factors perhaps play a certain role in the differences in thickness, hardness and leathery nature. It was reported that cellobiose, digalacturonic acid and gallic acid largely contributed to leaf thickness [38,39,40]. Gallic acid is the precursor of tannins, which are a class of more complex structural polyphenolic compounds in plants [40]. Cellobiose is only detected in *V. minor*, and it is the major metabolite affecting the formation of cell walls [38]. Cell walls not only maintain the shape of the plant cell types in different tissues and organs, but also play a very important role in plant growth and development [38,41]. Digalacturonic acid is a major component of pectin, promoting adhesion between cells and providing very important oligosaccharide molecule libraries and defense reactions [38,42]. Its accumulation will increase the leaf thickness and hardness by affecting the concentration of pectin [43]. These reports about the functions of the abovementioned three critical compounds are confirmed in our study, as we found that they were highly contained in the *V. minor* leaves, which displayed higher thickness and less leaf area compared with *C. roseus*.

*V. minor* is more resistant to cold, and its optimal growth environment temperature is 10 °C lower than that of *C. roseus* (Table 1). The smaller leaf size morphology, reflected in terms of LL, LW, LA, FW, LT, LMA, and the lower photosynthesis rate of *V. minor* will help plants adapt better to cold environments. It has been reported that the cold tolerance of plants is closely related to some carbohydrate metabolites [44]. Our result showed that raffinose and trehalose, which are recognized to promote plant resistance to cold conditions, were both largely accumulated in *V. minor* based on the GC-MS results. Trehalose is reported to help maintain a glassy structure under extreme temperatures and increases the stability of biomolecules [32]. The changes of raffinose level involve a series of fluctuations of raffinose family oligosaccharides (RFOS) metabolism (Table 2). RFOS metabolism is closely related to plant development and stress, and plays a key role in energy storage as a cryoprotectant against dehydration [44,45]. Raffinose can protect organelles from freezing damage, and it is more effective than other sugars during freeze–thaw cycles [46]. Furthermore, there is a frequent transport in the oligosaccharides, polyol and lipid transporter family in *V. minor*. Therefore, our results provide possible insights into the higher resistance of *V. minor* to cold damage since trehalose, raffinose and RFOS metabolites are present in greater amounts in this plant than in *C. roseus*.

It was interesting to find that leaf Pn was significantly higher in *V. minor*, while the energy pathway TCA cycle operated more efficiently in *C. roseus*. The energy flows from glucose ultimately toward to the metabolic pathway are associated with pyruvic acid, because pyruvic acid mainly moves towards the MEP pathway. We found that the content of glucose and pyruvic acid were higher in *C. roseus*. After entering the primary metabolic pathway, in *V. minor* loganin levels were 18- fold higher than in *C. roseus*. The pyruvic acid trend towards the pentose phosphate pathway, and the content of erythrose and tryptamine were higher in *C. roseus*. The intermediates tabersonine and serpentine from the upstream pathway of TIAs were both more enriched in *V. minor*. We speculate that the MEP pathway (loganin) plays a more important role than the pentose phosphate pathway in regulating the upstream secondary metabolism TIAs. However, the underlying mechanism of this induction process remains to be unraveled. Moreover, the level of eventual metabolite (vincamine) in *V. minor* was higher than in *C. roseus* (vinblastine) by about 2-fold. We conjecture that the reason for this result is that *V. minor* needs to invest more metabolic flux into defensive metabolism because it is a kind of vine plant vulnerable to be attack by soil-originating microorganisms.

In conclusion, we report a metabolomics comparison between *C. roseus* and *V. minor*, revealing the metabolic annotation for their biological and morphological divergence. We firstly use a non-targeted metabolomics method to identify a total of 58 significantly different metabolites classified into 16 sugars, 18 amino acids, nine alcohols and 18 organic acids, and 27 pathways were concentrated in the citric acid cycle, glycol metabolism, oligosaccharides, polyol and lipid transporter (RFOS) using GC-MS. Our results can explain why *V. minor* has smaller size, more leathery and is more resistant to cold than *C. roseus* based on leaf use of these different metabolites. Using a targeted metabolomics method directed analysis of nine TIAs by LC-MS. Metabolite network were drawn up to compare the differences of content and the distribution of classes of TIAs between *C. roseus* and *V. minor*. The networks revealed that the accumulation level of TIAs was higher in *V. minor* than *C. roseus*, and secondary metabolism synthesis was more regulated by the MEP pathway. Therefore, our research provides some basic data for further study on the leaves of *C. roseus* and *V. minor*. Based on the growth environment, leaf morphology characteristics, energy input and TIAs yields, *V. minor* might be better than *C. roseus* in the production of secondary metabolites.

## 4. Materials and Methods

### 4.1. Plant Materials

Well-grown *C. roseus* and *V. minor* plants were selected as study materials. Plant germination and growth was performed in a growth chamber (light period 8:00–22:00, under a 12/12 light/dark photoperiod at a temperature of 28 °C (day)/25 °C (night) and 65% relative humidity), where seedlings were irrigated with 1/2 strength Hoagland solution (pH 5.9–6.0). Leaf tissue samples of *C. roseus* and *V. minor* for GC-MS and LC-MS assays were taken during vegetative growth (10 June 2016). The fully expanded leaves at the top of the canopy were excised from individual plants and plunged immediately into liquid N_2_ and then stored at −80 °C for metabolite analysis. The GC-MS and LC-MS analysis was repeated six times for each species.

### 4.2. Leaf Morphology and Physiological Parameters

Fully expanded leaves were randomly selected for measurement of the physiological indicators. Leaf length (LL), leaf width (LW) and leaf area (LA) were measured by a leaf area meter (CI-203, CID, USA), and leaf thickness (LT) was measured by a plant leaf thickness tester (JZ-YHD, Boco Mai Instrument Co., Ltd., Ningbo, China). Fresh weight (FW), related water content (RWC) and leaf mass per unit area (LMA) were then calculated. Chlorophyll (Chl) was extracted from freshly sampled leaves using the acetone method of Porra [47]. Chl content was measured using a UV-VIS spectrophotometer (UV-5500 pc, Shanghai, China) Fluorescence parameters was measured using a fluorimeter (PAM-2500, Walz, Effeltrich, Germany). Leaves were kept in the dark for 25 min prior to the measurements. We refered to the method of Alfredo to measure and calculate the minimal (Fo), maximal (Fm), maximal variable fluorescence (Fv), ratio Fv/Fm and the practical quantum yield of PSII (Y(II)) [48]. Net photosynthesis rate (Pn), transpiration rate (Tr) and stomatal conductance (Gs) were evaluated using a LI-6400XT Portable Photosynthesis System (Li-Cor, Lincoln, NE, USA). Each replicate consisted of one measurement made on a fully expanded leaf per plant.

### 4.3. GC-MS Analysis

A collected leaf sample weighing 60 mg was mixed with 360 μL of cold methanol and 40 μL internal standard (0.3 mg/mL 2-chlorophenylalanine in methanol), and then homogenized using a tissuelyser system (Tissuelyser-192, Shanghai net letter technology companies, Shanghai, China). After ultrasonication for 30 min, 200 μL chloroform and 400 μL water were added to the sample. The mixture was vortexed for 2 min and sonicated for 30 min, and then the sample was centrifuged at 10,000× *g* for 10 min at 4 °C. Finally, 400 μL of the supernatant was transferred to a glass sampling vial for vacuum-drying at room temperature. The residue was derivatized using a two-step procedure. First, 80 μL methoxyamine (15 mg/mL in pyridine) was added to the vial, vortexed for 30 s and kept at 37 °C for 90 min followed by 80 μL BSTFA (1% TMCS) and 20 μL *n*-hexane at 70 °C for 60 min. After derivatization of the leaf tissue, 1 μL of solution was injected into an Agilent 7890A-5975C GC-MS system (Agilent Corporation, Santa Clara, CA, USA) with a split ratio of 30 to 1. Separation was carried out on a non-polar DB-5 capillary column (30 m × 250 μm I.D., J&W Scientific, Folsom, CA, USA), with high purity helium as the carrier gas at a constant flow rate of 1.0 mL/min. The temperature of injection and ion source was set to 260 °C and 230 °C, respectively. Electron impact ionization (−70 eV) at full scan mode (*m*/*z* 30–600) was used, with an acquisition rate of 20 spectrum/second in the MS setting. The QC sample was prepared by mixing aliquots of the tissues samples to be a pooled sample, and then analyzed using the same method with the analytic samples.

The acquired MS data from the GC-MS were analyzed by the ChromaTOF software (v 4.34, LECO, St. Joseph, MI, USA). Briefly, after alignment with the Statistic Compare module, a CSV file with three-dimension data sets including sample information, retention time and peak intensities was obtained. An internal standard was used for data quality control (reproducibility). Internal standards and any known pseudo positive peaks, such as peaks caused by noise, column bleed and the BSTFA derivatization procedure, were removed from the data set. The data set was normalized using the sum intensity of the peaks in each sample.

The data sets resulting from GC-MS were separately imported into SIMCA-P13 software package (Umetrics, Umea, Sweden). Principle component analysis (PCA) and partial least-squares-discriminant analysis (PLS-DA) were carried out to visualize the metabolic differences among experimental groups, after mean centering and unit variance scaling. All of the differentially expressed compounds in the treated group were selected by comparing the compounds in the treated group with the control using the multivariate statistical method, the Student’s *t*-test and Wilcoxon signed rank. Metabolites with both multivariate and univariate statistical significance (VIP > 1.0 and *p* < 0.05) were extracted. Those variables with VIP > 1.0 are considered as relevant for group discrimination. In this study, the default 7-round cross-validation was applied with 1/7 of the samples being excluded from the mathematical model in each round, in order to guard against overfitting. Then we get different metabolites by MBROLE website analyses. The KEGG pathway *p*-values were calculated via comparing with the proportion of metabolites in this pathway. Finally, the KEGG enrichment was used to illustrate pathway regulation and build model by plotting a histogram based on −log10.

### 4.4. LC-MS Analysis

A collected leaf sample (1.0 g) was freeze-dried in absolute methanol (analytical grade, 20 mL) for extraction of tryptamine, loganin, tabersonine, serpentine, vindoline, catharanthine, vincamine, vinblastine and vincristine. Low-frequency ultrasonication (250 W, 40 kHz) for 40 min was used to extract the alkaloids. The methanol extract was centrifuged at 8000 rpm for 10 min, concentrated to 2 mL, and the amount of alkaloids were determined by HPLC-MS (Ultra-performance LC, Waters, Tokyo, Japan; MS, AB SCIEX, USA) using an ACQUITY UPLC BEH C18 Column (1.7 µm, 2.1 mm × 50 mm) in an oven at 30 °C, with CH_3_CN/H_2_O and 0.05 moL L^−1^ ammonium acetate standard solvent system, respectively. The retention times of the alkaloids were 3.49 min (serpentine), 2.91 min (tabersonine), 3.37 min (vindoline), 3.32 min (vinblastine), 2.81 min (catharanthine), 0.76 min (loganin), 1.99 min (vincamine) and 2.49 min (vincristine). The sample injection volume was 10 μL at a flow rate of 1 mL/min. The TIAs content was quantified by tagged of the level from 10^−7^ to 10^−2^ mg L^−1^ based on peak area, respectively.

### 4.5. Statistical Analysis

Metabolite data were log2 transformed to improve normality and Min-Max Normalization. A total of two samples were used for hierarchical clustering analysis by R to study the variations of *C. roseus* and *V. minor* in leaves. The heat maps, histogram and pathway maps to display the experimental data structure were drawn with R-3.2 language software, Sigmaplot-10.0 and Visor, respectively.

## Figures and Tables

**Table 1:** The morphological indicators and photosynthetic parameters of the leaves of *V. minor* and *C. roseus*.

Indicator/Parameter	*C. roseus*	*V. minor*
LL (cm)	5.08 ± 0.37	2.36 ± 0.25
LW (cm)	2.50 ± 0.21	1.41 ± 0.19
LA (cm^2^)	8.42 ± 0.86	2.67 ± 0.41
FW (g)	0.15 ± 0.010	0.07 ± 0.0087
LT (cm)	0.115 ± 0.012	0.19 ± 0.018
RWC (%)	88.67 ± 7.32	51.43 ± 3.81
LMA (g/cm^2^)	0.018 ± 0.0021	0.027 ± 0.0013
Pn (μmol CO_2_ m^−2^ s^−1^)	8.03 ± 0.23	11.67 ± 0.50
Gs (mol H_2_O m^−2^ s^−1^)	0.042 ± 0.0002	0.038 ± 0.0008
Tr (mmol H_2_O m^−2^ s^−1^)	1.56 ± 0.0164	1.49 ± 0.016
Fv/Fm	0.815 ± 0.0083	0.841 ± 0.0084
Y (II)	0.711 ± 0.0199	0.761 ± 0.0112
Chl (mg/g)	11.23 ± 2.54	7.93 ± 1.65
Chla/chlb	4.17 ± 0.55	3.34 ± 0.36
Temperature (°C)	20–33	15–25

LL, leaf length; LW, leaf width; LA, leaf area; FW, fresh weight; LT, leaf thickness; RWC, relative water content; LMA, leaf mass per unit area; Pn, net photosynthesis rate; Gs, Stomatal conductance; Tr, Transpiration rate; Chl, chlorophyll; Chla/chlb, chlorophylla/chlorophyllb; average ± standard deviation.

**Table 2:** The different metabolites between *V. minor* and *C. roseus*.

KEGG (*p*-Value)	Different Metabolites	Vip	Change Fold (*V.* *minor*/*C.* *roseus*)	*p*-Value
Galactose metabolism (4.74 × 10^−4^)	Raffinose #	1.06	——	** ——
	Myo-inositol ##	1.24	0.13	** 0.005
	Galactinol #	1.42	0.19	** 9.7 × 10^−6^
	Fructose #	1.28	0.03	** 0.004
	Glucose #	1.10	0.18	* 0.020
TCA cycle (7.31 × 10^−4^)	Fumaric acid ###	1.28	0.09	** 0.004
	alpha-ketoglutaric acid ###	1.38	0.02	** 0.0009
	Pyruvic acid ###	1.47	0.09	** 3.3 × 10^−7^
Starch and sucrose metabolism (1.02 × 10^−3^)	Trehalose #	1.06	77.64	* 0.029
	Cellobiose #	1.09	——	** ——
	Fructose			
	Glucose			
Oligosaccharides, polyol and lipid transporter (3.45 × 10^−2^)	Raffinose			
	Mannitol ##	1.25	0.13	** 0.004
	Trehalose			
	Cellobiose			
Biosynthesis of phenylpropanoids (1.70 × 10^−3^)	alpha-ketoglutaric acid			
	Salicin #	1.10	5.26	* 0.018
	Gallic acid ###	1.01	3.02	* 0.019
	Pyruvic acid			
	Fumaric acid			
Biosynthesis of terpenoids and steroids (1.17 × 10^−2^)	Fumaric acid			
	Pyruvic acid			
	alpha-ketoglutaric acid			
	Loganin #	1.48	18.19	** 3.4 × 10^−5^
Glyoxylate and dicarboxylate metabolism (6.24 × 10^−4^)	alpha-ketoglutaric acid			
	Pyruvic acid			
	Glycolic acid ###	1.49	0.05	** 1.2 × 10^−8^
Cyanoamino acid metabolism (4.74 × 10^−4^)	Tartaric acid ###	1.08	6.19	* 0.023
	Glutamic acid ####	1.30	0.08	** 0.003
	4-Hydroxymandelonitrile	1.26	4.87	** 0.004
	Serine ####	1.12	52.77	* 0.022
	Tyrosine ####	1.14	0.18	* 0.012
Butanoate metabolism (3.63 × 10^−2^)	Pyruvic acid			
	α-Ketoglutaric acid			
	Fumaric acid			
	Maleic acid ###	1.02	0.21	* 0.017
Biosynthesis of alkaloids derived from shikimate pathway (9.31 × 10^−3^)	Tyrosine			
	Fumaric acid			
	Pyruvic acid			
	α-Ketoglutaric acid			
Biosynthesis of alkaloids derived from terpenoid and polyketide (9.31 × 10^−3^)	α-Ketoglutaric acid			
	Fumaric acid			
	Pyruvic acid			
Pentose phosphate pathway (2.95 × 10^−3^)	Glucose			
	Pyruvic acid			
	Gluconic lactone #	1.11	0.16	** 0.0076
Glycolysis/Gluconeogenesis (2.69 × 10^−3^)	Salicin #			
	Glucose			
	Pyruvic acid			
C5-Branched dibasic acid metabolism (2.95 × 10^−3^)	Itaconic acid ###	1.00	5.45	* 0.02
	α-Ketoglutaric acid			
	Pyruvic acid			
Ascorbate and aldarate metabolism (8.78 × 10^−3^)	α-Ketoglutaric acid			
	Pyruvic acid			
	Myo-inositol			
Biosynthesis of alkaloids derived from histidine and purine (3.82 × 10^−3^)	α-Ketoglutaric acid			
	Pyruvic acid			
	Fumaric acid			
Biosynthesis of plant hormones (2.38 × 10^−2^)	α-Ketoglutaric acid			
	Pyruvic acid			
	Fumaric acid			
Phenylalanine metabolism(8.27 × 10^−3^)	Tyrosine			
	Pyruvic acid			
	Fumaric acid			
Nicotinate and nicotinamide metabolism (7.31 × 10^−3^)	Pyruvic acid			
	Fumaric acid			
	Maleic acid ###			
Tyrosine metabolism(3.18 × 10^−2^)	Pyruvic acid			
	Fumaric acid			
	Tyrosine			
Glycine, serine and threonine metabolism (9.85 × 10^−3^)	Pyruvic acid			
	L-Allothreonine ####	1.46	0.01	** 7.9 × 10^−5^
	Serine ####	1.12	52.77	* 0.022
Biosynthesis of alkaloids derived from ornithine, lysine and nicotinic acid (2.29 × 10^−2^)	Pyruvic acid			
	Fumaric acid			
	α-Ketoglutaric acid			
Alanine, aspartate and glutamate metabolism (1.26 × 10^−3^)	α-Ketoglutaric acid			
	Pyruvic acid			
Oxidative phosphorylation(9.13 × 10^−3^)	Phosphate	1.33	0.07	** 0.002
	Fumaric acid			
Thiamine metabolism(2.34 × 10^−2^)	Pyruvic acid			
	Tyrosine			
Phenylalanine, tyrosine and tryptophan biosynthesis (2.51 × 10^−2^)	Quinic acid ###	1.17	3.28	** 0.004
	Tyrosine			
Taurine and hypotaurine metabolism (1.41 × 10^−2^)	2-Aminoethanethiol	1.14	0.19	* 0.010
	Pyruvic acid			
Others	Tagatose #	1.30	0.05	** 0.003
	Levoglucosan #	1.23	11.66	** 0.006
	Erythrose #	1.13	0.44	** 0.006
	Lyxose #	1.13	3.02	** 0.005
	Ribose #	1.34	0.13	** 0.0002
	d-Talose#	1.19	0.03	* 0.011
	3,6-Anhydro-d-galactose #	1.29	0.24	** 0.0005
	Naringin #	1.08	0.35	** 0.0098
	Glucoheptonic acid ###	1.20	130715.28	** 0.0096
	Gluconic acid ###	1.17	15.46	* 0.013
	Chlorogenic Acid ###	1.35	0.21	** 0.0002
	Threonic acid ###	1.16	21.18	* 0.014
	d-Glyceric acid ###	1.15	4.95	* 0.012
	Galactonic acid ###	1.02	19.60	* 0.040
	Aminooxyacetic acid ###	1.29	4.30	** 0.0004
	Digalacturonic acid ###	1.11	101.91	* 0.023
	3-Aminoisobutyric acid ###	1.29	32.74	** 0.005
	Dodecanol ##	1.16	0.46	** 0.004
	Lactitol ##	1.13	6.12	* 0.016
	Threitol ##	1.07	7.92	* 0.025
	3-Methylamino-1,2-propanediol ##	1.04	0.06	* 0.029
	1,5-Anhydroglucitol ##	1.21	198.25	** 0.009
	Phytol ##	1.13	0.06	* 0.015
	2-Amino-1-phenylethanol ##	1.10	0.16	* 0.018
	Threonine ####	1.31	5.26	** 0.0004
	Aspartic acid ####	1.44	0.11	** 8.6 × 10^−5^
	Ornithine ####	1.25	0.23	** 0.001
	Valine ####	1.19	0.18	** 0.003
	2-Kydroxypyridine	1.41	0.11	** 1.4 × 10^−5^
	Octanal	1.05	——	** ——
	2-aminoethanethiol	1.14	0.19	* 0.010
	Gluconic lactone	1.11	0.16	** 0.0076
	Glutamine	1.08	0.01	* 0.024

VIP, variable importance in the projection; Significant * *p* < 0.05, Extremely significant ** *p* < 0.01, sugars were represented by #, alcohol were represented by ##, acids were represented by ###, amino acids were represented by ####.

## Supplementary materials

Supplementary materials are available online.
