# Supplementary material for: Metabolomics Characterization of Two Apocynaceae Plants, Catharanthus roseus and Vinca minor, Using GC-MS and LC-MS Methods in Combination
Source: Molecules. 2017 Jun 17;22(6):997. doi: 10.3390/molecules22060997 (PMC6152753; doi:10.3390/molecules22060997)
Supplement: Supplementary file 1 [file molecules-22-00997-s001.pdf]

Supplementary Materials

Table S1. Pearson correlation matrix of trehalose and five physiological indicators in *C. roseus.*

	Trehalose	LL	LW	LA	FW
Trehalose	1	−0.441	−0.527	−0.032	−0.134
LL		1	0.832*	−0.651	−0.027
LW			1	−0.718	0.152
LA				1	−0.469
FW					1

LL, leaf length; LW, leaf width; LA, leaf area; FW, fresh weight; *$p < 0.05$; **$p < 0.01$; ***$p < 0.001$.

Table S2. Pearson correlation matrix of trehalose and five physiological indicators in *V. minor.*

	Trehalose	LL	LW	LA	FW
Trehalose	1	−0.38	−0.609	−0.845*	−0.354
LL		1	0.666	0.484	−0.027
LW			1	0.542	0.174
LA				1	−0.075
FW					1

LL, leaf length; LW, leaf width; LA, leaf area; FW, fresh weight; *$p < 0.05$; **$p < 0.01$; ***$p < 0.001$.

Table S3. Pearson correlation matrix of digalacturonic acid and two physiological indicators in *C. roseus.*

	Digalacturonic Acid	LA	LT
Digalacturonic Acid	1	0.34	0.244
LA		1	0.294
LT			1

LA, leaf area; LT, leaf thickness; *$p < 0.05$; **$p < 0.01$; ***$p < 0.001$.

Table S4. Pearson correlation matrix of gallic acid and tree physiological indicators in *C. roseus.*

	Gallic Acid	LW	LT	LMA
Gallic Acid	1	0.298	0.169	0.673
LW		1	−0.555	0.588
LT			1	−0.283
LMA				1

LW, leaf width; LT, leaf thickness; LMA, leaf mass per unit area; *$p < 0.05$; **$p < 0.01$; ***$p < 0.001$.

Table S5. Pearson correlation matrix of tree metabolites and leaf thickness in *V. minor.*

	LT	Cellobiose	Digalacturonic Acid	Gallic Acid
LT	1	0.059	0.498	0.743
Cellobiose		1	−0.219	0.247
Digalacturonic acid			1	0.726
Gallic acid				1

LT, leaf thickness; *$p < 0.05$; **$p < 0.01$; ***$p < 0.001$.

Table S6 Pearson correlation matrix of raffinose and tree physiological indicators in *V. minor.*

	**Raffinose**	**RWC**	**Tr**	**LT**
Raffinose	1	−0.025	−0.479	0.725
RWC		1	0.524	−0.253
Tr			1	−0.643
LT				1

RWC, relative water content; Transpiration rate, Tr; LT, leaf thickness; *p < 0.05; **p < 0.01; ***p < 0.001.

Table S7. The content of TIAs in the leaf of *C. roseus* and *V. minor*.

**TIA**	***C. roseus*** **(mg g^{-1})**	***V. minor*** **(mg g^{-1})**
Loganin	0.043 ± 0.01	0.756 ± 0.11
Tryptamine	0.007 ± 0.0004	0.002 ± 8 × 10^{-5}
Tabersonine	0.001 ± 0.0002	0.003 ± 0.0001
Serpenitine	0.008 ± 0.0015	0.044 ± 0.005
Catharanthine	0.0004 ± 0.0002	--
Vindoline	0.051 ± 0.01	--
Vinblastine	0.093 ± 0.007	--
Vincristine	0.067 ± 0.016	--
Vinacmine	--	0.173 ± 0.04

The calculation of FDR was based on the following formula: p-value(i) = p(i)* length(p)/rank(p).

Table S8. The FDR result of different metabolites between *C. roseus* and *V. minor*.

**Metabolites**	**FDR**
Glycolic acid	7.97143 × 10^{-7}
Pyruvic acid	1.0603 × 10^{-5}
Galactinol	0.0002
2-Hydroxypyridine	0.0002
Loganin	0.0004
L-Allothreonine	0.0008
Aspartic acid	0.0008
Chlorogenic Acid	0.001
Ribose	0.001
Threonine	0.002
Aminooxyacetic acid	0.003
3,6-Anhydro-D-galactose	0.003
α-Ketoglutaric acid	0.005
Ornithine	0.005
Phosphate	0.008
Valine	0.012
Glutamic acid	0.012
Tagatose	0.012
4-Hydroxymandelonitrile	0.012
Fumaric acid	0.012
Dodecanol	0.011
Quinic acid	0.011
Mannitol	0.011
Fructose	0.012
3-Aminoisobutyric acid	0.012
myo-Inositol	0.012

Lyxose	0.013
Erythrose	0.013
Levoglucosan	0.014
Gluconic lactone	0.016
1,5-Anhydroglucitol	0.02
Glucoheptonic acid	0.019
Naringin	0.019
2-Aminoethanethiol	0.019
D-Talose	0.02
D-Glyceric acid	0.02
Tyrosine	0.02
Gluconic acid	0.02
Threonic acid	0.02
Phytol	0.02
Lactitol	0.02
Maleic acid	0.02
Salicin	0.03
2-Amino-1-phenylethanol	0.03
Gallic acid	0.03
Glucose	0.03
Itaconic acid	0.03
Serine	0.03
Digalacturonic acid	0.03
Tartaric acid	0.03
Glutamine	0.03
Threitol	0.03
Trehalose	0.04
3-Methylamino-1,2-propanediol	0.03
Galactonic acid	0.046
Oxoproline	--
Vanillylmandelic acid	--
N-Acetyl-L-glutamic acid	--
4-Hydroxybutyrate	--
Cellobiose	--
Raffinose	--
Octanal	--
Norleucine	--
Galactose	--

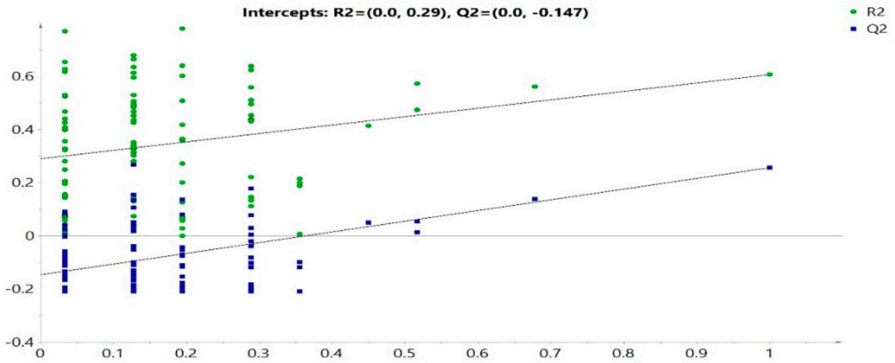

Figure S1 Validate Model of PLS-DA score plot between *Catharanthus roseus* and *Vinca minor*.

Table S9. The CV-ANOVA of PLS-DA.

PLS-DA	SS	DF	MS	F	p	SD
Total corr.	11	11	1			1
Regression	10.6764	4	2.6691	57.7405	1.92×10^{-5}	1.63374
Residual	0.323581	7	0.046226			0.215002

SS, sum of square, DF, degree of freedom, MS, Mean of aquare, f-statistics, P,P-vaule, SD, mean square error.

Table S10. P-values of metabolites between *C. roseus* and *V. minor*.

Peak	Change Fold (*V. minor*/ *C. roseus*)	F-Test	T-Test Equal Variance	T-Test Heteroscadasticity	P-Value
Valine	0.375072	0.115696	0.002997	0.005882	0.002997
Tyrosine	0.60188	0.032613	0.005474	0.011989	0.011989
Threonine	3.504796	0.171639	0.000404	0.001024	0.000404
Threonic acid	9.250436	1.18×10^{-6}	0.004328	0.014382	0.014382
Tartaric acid	2.405982	0.001024	0.009759	0.022807	0.022807
Tagatose	0.070212	0.000201	0.000436	0.003375	0.003375
Serine	38.14592	3.33×10^{-7}	0.008391	0.022067	0.022067
Saccharic acid	3.322235	1.36×10^{-5}	0.025967	0.047175	0.047175
Quinic acid	2.766339	0.141575	0.003915	0.006904	0.003915
Proline	0.587889	0.015704	0.347234	0.363233	0.363233
Phosphate	0.288326	0.000398	0.00018	0.001962	0.001962
Oxalic acid	0.996282	0.370714	0.982956	0.983024	0.982956
Ornithine	0.599397	0.950994	0.001058	0.00106	0.001058
Nicotinoylglycine	0.836692	0.127196	0.136525	0.148644	0.136525
myo-Inositol	0.160401	0.020035	0.001307	0.004677	0.004677
Monophthalate	1.176763	0.98026	0.215181	0.215185	0.215181
Methyl phosphate	0.855084	0.803597	0.221122	0.221507	0.221122
Methoxamedrine	1.332328	0.472711	0.057474	0.060356	0.057474
Mannitol	0.134895	0.019075	0.001059	0.004086	0.004086
Maleic acid	0.492775	0.070903	0.01736	0.026358	0.01736
Lyxose	2.383039	0.166572	0.005404	0.00863	0.005404
Loganin	12.1263	0.001066	1.36×10^{-7}	3.37×10^{-5}	3.37×10^{-5}
L-Malic acid	0.943323	0.890616	0.708529	0.708562	0.708529
Lactitol	2.776218	0.001441	0.006073	0.016375	0.016375
Lactic acid	0.889478	0.198464	0.412545	0.418461	0.412545
Kyotorphin	0.984454	0.99196	0.898954	0.898954	0.898954
Itaconic acid	4.161146	0.258221	0.020405	0.024892	0.020405
Glycerol	0.756464	0.003352	0.347434	0.366145	0.366145
Glutamic acid	0.18961	0.000982	0.000441	0.003179	0.003179
Glucose-1-phosphate	22.92819	2.55×10^{-10}	0.098665	0.128288	0.128288

Table S10. P-values

**Glucose**	**0.292022**	**0.00542**	**0.009101**	**0.02032**	**0.02032**
Gluconic acid	3.971332	6.68×10^{-5}	0.003719	0.012756	0.012756
Gallic acid	2.655112	0.969221	0.018595	0.018601	0.018595
Galactonic acid	12.21658	5.87×10^{-6}	0.020251	0.039734	0.039734
Galactinol	0.166298	0.071481	9.68×10^{-6}	0.000107	9.68×10^{-6}
Fumaric acid	0.188414	0.000858	0.000594	0.003834	0.003834
Fructose	0.052961	4.19×10^{-5}	0.000639	0.004378	0.004378
Ethanolamine	1.30358	0.436874	0.073022	0.076575	0.073022
Erythrose	0.492896	0.074069	0.005854	0.01104	0.005854
D-Talose	0.042032	3.2×10^{-6}	0.002917	0.011197	0.011197
Digalacturonic acid	19.0092	1.43×10^{-9}	0.00866	0.022586	0.022586

D-Glyceric acid	2.43669	0.010589	0.004413	0.011686	0.011686
D-Erythrosphingosine	1.10768	0.523602	0.872883	0.873159	0.872883
Creatine degr.	0.832335	0.125484	0.68469	0.688164	0.68469
Chlorogenic acid	0.222861	0.590343	0.000152	0.000193	0.000152
Cellobiotol	0.830813	0.032575	0.426183	0.437943	0.437943
β-Mannosylglycerate	0.79278	0.022095	0.664065	0.670369	0.670369
Aspartic acid	0.202807	0.033945	3.78×10^{-6}	8.65×10^{-5}	8.65×10^{-5}
Aminooxyacetic acid	1.963728	0.784327	0.000439	0.000462	0.000439
Alanine	0.451731	0.294491	0.028969	0.033532	0.028969
4-Androsten-11β-ol-3,17-dione	0.910629	0.093431	0.417119	0.425925	0.417119
4-Aminobutyric acid	1.064839	0.082951	0.738843	0.742185	0.738843
3-Aminoisobutyric acid	11.43573	1.66×10^{-6}	0.000673	0.004636	0.004636
3,6-Anhydro-D-galactose	0.325319	0.077758	0.000482	0.001652	0.000482
2-Aminoethanethiol	0.48208	0.043886	0.00471	0.010256	0.010256
2-Amino-1-henylethanol	0.349126	0.004492	0.007827	0.018483	0.018483
2,3-Dihydroxybenzoic acid	2.070183	0.001019	0.091386	0.117994	0.117994
Salicin	5.258054	0.014679	0.00834	0.017801	0.017801
Pyruvic acid	0.094812	0.10636	3.31×10^{-7}	7.25×10^{-6}	3.31×10^{-7}
Isopropyl-β-D-thiogalactopyranoside	0.580475	0.769039	0.165654	0.166222	0.165654
Isoleucine	3.455346	0.004353	0.069392	0.092842	0.092842
Glutaconic acid	0.041918	7.51×10^{-9}	0.331859	0.354583	0.354583
Dodecanol	0.463981	0.878177	0.003877	0.003912	0.003877
Caffeic acid	0.558169	0.002354	0.086198	0.111531	0.111531
4-Hydroxymandelonitrile	4.873474	0.014825	0.000864	0.003713	0.003713
Trehalose	77.64279	1.16×10^{-8}	0.012769	0.029204	0.029204
Spermidine	0.425786	0.531741	0.044842	0.046891	0.044842
Ribose	0.133108	0.050685	0.000196	0.00101	0.000196
Naringin	0.350139	0.569729	0.009802	0.010638	0.009802
5-methoxytryptamine	1.188753	0.012578	0.584299	0.593242	0.593242
Xylitol	0.695128	0.52482	0.542977	0.544157	0.542977
Threitol	7.917954	0.00524	0.011967	0.024701	0.024701
Methyl-beta-D-galactopyranoside	2.521722	0.006724	0.274831	0.295185	0.295185
Levoglucosan	11.66117	0.003168	0.001501	0.0063	0.0063
Fucose	0.672963	0.939881	0.315273	0.315304	0.315273
Citramalic acid	1.226498	0.744367	0.625368	0.625617	0.625368
2-hydroxypyridine	0.11099	0.170244	1.43×10^{-5}	7.45×10^{-5}	1.43×10^{-5}
2,3-dimethylsuccinic acid	0.467706	0.077846	0.207718	0.221826	0.207718
Glutamine	0.013499	6.38×10^{-7}	0.009773	0.024374	0.024374
Digitoxose	1.045652	0.712406	0.936379	0.936426	0.936379
Succinic acid	3.174374	0.046992	0.068619	0.084933	0.084933
Lysine	0.368187	0.067612	0.142529	0.158186	0.142529
L-allothreonine	0.010395	0.000251	5.17×10^{-7}	7.99×10^{-5}	7.99×10^{-5}
Glycolic acid	0.048918	0.282002	1.25×10^{-8}	1.35×10^{-7}	1.25×10^{-8}
Gluconic lactone	0.160577	0.686052	0.007613	0.007981	0.007613
Glucoheptonic acid	130715.3	3.19×10^{-22}	0.002238	0.009603	0.009603
α-Ketoglutaric acid	0.015797	0.00017	4.56E-05	0.000931	0.000931
4-Hydroxyquinazoline	0.498172	0.590227	0.237791	0.239508	0.237791
1,5-Anhydroglucitol	198.2445	4.11×10^{-8}	0.002216	0.009528	0.009528
Vanillylmandelic acid	--	--	0.000194	0.00229	--
Raffinose	--	--	0.011874	0.027836	--
Phytol	0.057049	0.004321	0.005861	0.015181	0.015181
Oxoproline	0	--	6.42×10^{-8}	3.25×10^{-5}	--
Maleamate	6.684093	0.034226	0.09586	0.114428	0.114428
3-Methylamino-1,2-propanediol	0.062704	0.003072	0.01476	0.029353	0.029353
Phenyl β-D-glucopyranoside	--	--	0.056775	0.083925	--
Octanal	--	--	0.015752	0.033673	--
N-acetyl-L-glutamic acid	0	--	0.001731	0.008213	--
Isocitric acid	0	--	0.026588	0.048373	--

Glycocyamine	4.339811	0.012596	0.195567	0.216914	0.216914
Cellobiose	--	--	0.009337	0.023737	--
6-Methylmercaptopurine	1.545968	0.816308	0.577346	0.577494	0.577346
4-Hydroxybutyrate	--	--	0.002026	0.009035	--
3-Hydroxypropionic acid	1.425287	0.109492	0.701794	0.705267	0.701794
1-Hydroxyanthraquinone	1.922166	0.53409	0.411057	0.412657	0.411057
Phytosphingosine	200.3828	1.07×10^{-9}	0.07473	0.103355	0.103355
Norleucine	0	--	0.016703	0.035044	--
Methionine	2.874531	0.47303	0.302624	0.30528	0.302624
Maltotriose	--	--	0.022353	0.042836	--
L-Dopa	--	--	0.028393	0.050671	--
Glucose-6-phosphate	0.079847	0.000964	0.103309	0.130271	0.130271
Gentiobiose	--	--	0.040014	0.064823	--
Galactose	0	--	0.024115	0.045167	--
4-Methylbenzyl alcohol	3.233402	0.376505	0.248543	0.252847	0.248543
Sorbitol	--	--	0.068307	0.096502	--
